# Epidemiological Characteristics of Overseas-Imported Infectious Diseases Identified through Airport Health-Screening Measures: A Case Study on Fuzhou, China

**DOI:** 10.3390/tropicalmed9060138

**Published:** 2024-06-20

**Authors:** Hong Li, Yan Yang, Jiake Chen, Qingyu Li, Yifeng Chen, Yilin Zhang, Shaojian Cai, Meirong Zhan, Chuancheng Wu, Xinwu Lin, Jianjun Xiang

**Affiliations:** 1 School of Public Health, Fujian Medical University, Fuzhou 350122, China; kinglh2002@126.com (H.L.); yy18085245362@163.com (Y.Y.); 18598903455@163.com (J.C.); 15700767080@163.com (Q.L.); 18059714167@163.com (Y.C.); ayilin678@163.com (Y.Z.); wcc@fjmu.edu.cn (C.W.); 2 Key Laboratory of Environment and Health, Fujian Province University, Fuzhou 350122, China; 3 School of Public Health and Health Management, Fujian Health College, Fuzhou 350101, China; 4 Department of Emergency Preparedness and Response, Fujian Provincial Center for Diseases Control and Prevention, Fuzhou 350012, China; lbcsjbe@163.com (S.C.); zmr@fjcdc.com.cn (M.Z.); 5 Entry Health Screening Office, Fuzhou Customs, Changle International Airport, Fuzhou 350209, China; 6 School of Public Health, The University of Adelaide, North Terrace Campus, Adelaide, SA 5005, Australia

**Keywords:** border health screening, imported infection, epidemiology, travel-associated

## Abstract

Background: This study aimed to examine the epidemiological characteristics of imported infections and assess the effectiveness of border health screening in detecting imported diseases. Methods: We obtained infection data for 2016 to 2019 from the Fuzhou Changle International Airport Infection Reporting System. The demographic, temporal, and spatial characteristics of travel-related infections were analyzed using r×c contingency tables, the Cochran–Armitage trend test, and seasonal-trend decomposition using LOESS (STL). Detection rates were used as a proxy for the effectiveness of border health-screening measures. Results: Overall, 559 travel-related infections were identified during the study period, with 94.3% being imported infections. Airport health screening demonstrated an overall effectiveness of 23.7% in identifying travel-associated infections. Imported infections were predominantly identified in males, with 55.8% of cases occurring in individuals aged 20–49. The peak periods of infection importation were from January to February and from May to August. The infectious diseases identified were imported from 25 different countries and regions. All dengue fever cases were imported from Southeast Asia. Most notifiable infections (76.0%) were identified through fever screening at the airport. Conclusion: The increasing number of imported infections poses a growing challenge for public health systems. Multifaceted efforts including surveillance, vaccination, international collaboration, and public awareness are required to mitigate the importation and spread of infectious diseases from overseas sources.

## 1. Introduction

China was the world’s fourth most popular destination before the COVID-19 pandemic, with 66 million incoming visitors in 2019, accounting for 4.5% of global travelers, and air travel is the dominant means of cross-border traveling (corresponding to 60%) [1]. Huge increases in international travel and trade activities allow disease agents to spread quickly from one side of the world to another within a couple of hours, increasing the risk of outbreaks evolving into epidemics or pandemics. The types of infectious diseases imported into mainland China significantly increased from 2 in 2005 to 45 in 2016 [2].

A total of 17,189 travelers were diagnosed with 58 types of imported infectious diseases from 2014 to 2018, with an incidence rate of 122.59 per million inbound travelers [3]. Moreover, these numbers are thought to be underestimated, as some pathogens carried by the travelers may have been in the incubation period at the time of arrival and missed by the border health-screening system [4]. The increasing integration of China into the regional/global economy, as exemplified by the Belt and Road Initiative, may amplify the risk of infectious disease importation and further burden the already-overloaded healthcare systems [5].

In recognition of the need to reconcile global public health efforts against emerging and re-emerging infectious diseases, the World Health Organization revised the International Health Regulations in 2005. To address the risk of importation-driven autochthonous transmission, the General Administration of Quality Supervision, Inspection, and Quarantine of China required personnel at all border points of entry to conduct active surveillance (i.e., entry health screening) of infectious diseases among incoming passengers starting in 2014 [6]. The entry-screening approaches include fever screening, medical inspection, self-declaration, and reporting by on-board staff, aiming to prevent or delay the importation of diseases or incubating cases to the country. There are some studies attempting to assess the effectiveness of border entry screening measures, although these findings are inconsistent. Nevertheless, most of the published literature suggests that the effectiveness of entry health-screening measures is very limited in terms of detecting imported cases at borders [7,8].

Evidence has shown that all provinces and municipalities in China have reported imported infectious diseases so far [2], and most cases were imported into coastal provinces of the eastern and southeastern regions [6]. Fuzhou is the capital city of Fujian Province on the southeastern coast of China, and it is also a renowned hometown of overseas Chinese. Over 3 million overseas Chinese originate from Fuzhou, which means imported infectious diseases may have a huge impact on this city. In this study, we analyzed the epidemiological characteristics of imported infections identified through entry-screening measures at Fuzhou International Airport; and utilized a data linkage approach to assess the effectiveness of entry-screening measures. Using Fuzhou as a case, the findings of this study may provide useful information for the improvement of border health-screening practices nationally and internationally to minimize the risk of importation-triggered disease outbreaks.

## 2. Materials and Methods

### 2.1. Surveillance System of Imported Infectious Diseases

Currently, there are two main databases for the surveillance of imported infectious diseases in China. The active surveillance system includes the afore-mentioned fever screening at the point of entry, the collection of health statements from the inbound passengers, medical inspections, and reporting by onboard staff [6]. The passive surveillance system refers to the China National Notifiable Disease Surveillance System (NDSS), which was launched in 2004 and is hierarchically managed by different levels of the Centers for Disease Control and Prevention (CDC). There are 40 notifiable infectious diseases, which are divided into three classes, namely, A (2), B (26, including COVID-19), and C (12), in descending order according to severity. Category-A infectious diseases are required to be reported online to the NDSS within 2 h after diagnosis, and the other two categories of infectious diseases must be reported within 24 h [9].

### 2.2. Data Collection

Fuzhou Changle International Airport (FOC) is a major transportation hub in the south-east region of China. FOC’s handling capacity reached approximately 15 million passengers in 2019 [10]. More than 50 airlines operate from this airport, with flights to destinations in Southeast Asia, Europe, Oceania, and North America. De-identified entry-and-exit health-screening data and relevant variables for the period from 1 January 2016 to 31 December 2019 were obtained from the Entry Health-Screening Office, Fuzhou Customs. Relevant variables include age, gender, nationality, itinerary details (i.e., place and date), clinical symptoms, case-finding approach, means of specimen collection, onset date of disease, and diagnosis-related information. Corresponding imported notifiable infectious diseases reported through the NDSS for Fujian Province were obtained from Fujian Provincial CDC during the same study period. This study was approved by the Ethics Committee of Fujian Medical University.

There are standardized operation procedures (e.g., fever screening, medical inspection, self-declaration, and reporting by on-board staff) at each entry point to identify suspected cases when incoming international travelers pass through customs [6]. The alert threshold of fever screening is usually set as 37.8 °C. Suspected travelers are interviewed by health quarantine staff using a standardized questionnaire to collect demographics, itinerary information, and clinical manifestations. Moreover, according to the suspected type of infection, biological specimens such as nasopharyngeal swabs, blood, urine, sputum, feces, vomitus, and serum samples are collected to conduct rapid tests on-site or sent to the local surveillance center for testing, if necessary. Those with positive results are subjected to relevant quarantine/isolation measurements and/or transferred for medical treatment, depending on the severity and type of infection. Meanwhile, confirmed cases are required to be reported to the NDSS system to inform the local CDC. Close contacts are informed of measures for prevention, quarantine, or isolation.

### 2.3. Statistical Analysis

In this study, imported infections were classified into three types: respiratory, gastrointestinal, and vector-borne. Cases without specific diagnoses or travel itineraries were excluded from the analysis. To assess the effectiveness of entry health-screening measures, we linked the imported notifiable infectious diseases identified at the entry point with the data extracted from the NDSS reporting system, using a unique identifier in this process (i.e., passport number). If passport numbers were not available, the two datasets were linked by other identifiers such as gender, age, and nationality.

The demographic characteristics of travel-related infections were descriptively analyzed. Associations between disease types and the independent variables (gender, age, citizenship, case-finding approach, and clinical symptoms) were explored by using r × c contingency tables. The conditional probabilities (column percentages; tabulate command) and adjusted residuals (tabchi command) were reported. Any cells with adjusted residuals greater than ±1.96 were highlighted in bold, as they represent more extreme cases than would be expected if the null hypothesis of independence was true [11].

Detection rates were used as proxy for effectiveness of border health-screening measures. The entry detection rate was calculated as the number of cases successfully identified at the point of entry divided by the total number of suspected incoming passengers. The overall detection rate (entry and exit) was calculated as the number of cases confirmed at the border divided by the total number of suspected inbound and outbound travelers. Meanwhile, seasonal-trend decomposition procedure based on LOESS (STL) [12] was used to decompose a time series into seasonal, trend, and remainder components using LOESS. The time series data, seasonal component, trend-cycle component, and remainder component were denoted by $Y_{t}$, $S_{t}$, $T_{t}$, and $R_{t},$ respectively, for t = 1 to N. And the equation used was $Y_{t}=S_{t}+T_{t}+R_{t}$. Cochran–Armitage trend test was used to examine the trend of imported notifiable infections over time [13]. The seasonal index was applied to describe the seasonal fluctuation of imported infections. The index for a given month was calculated by dividing the average case number of the month in question by the average monthly cases over 4 years (2016–2019). Seasonal fluctuation is not significant if the index in each month is close to 1 [14]. All statistical analyses were performed using R version 4.2.2 (R Core Team, Vienna, Austria), Excel 365 (Microsoft, Redmond, WA, USA), GraphPad Prism (version 9.0.0 La Jolla, CA, USA), and ArcGIS (version 10.8, Redlands, CA, USA).

## 3. Results

### 3.1. Demographic Characteristics

As shown in Table 1, during the 2016–2019 period, 2362 passengers were identified as suspected infected individuals via the border health-screening measures at FOC Airport, among which 559 (23.7%) travelers were confirmed to have infections. Among the cases, 527 were imported infections (94.3%). The 2362 suspected cases included 2249 inbound travelers, with an entry detection rate of 23.4%. For both the suspected (63.8%) and confirmed cases (66.0%), males accounted for about two-thirds. The vast majority (83.2%) of confirmed infections were found in individuals less than 50 years old, with an interquartile range of 29 (0–79). Travelers of mainland China accounted for 85.3% of the confirmed infections. In terms of the type of infections, 88.7% were respiratory, while vector-borne infections constituted 8.6%, and gastrointestinal infections constituted 2.7%. Imported gastrointestinal infections were more likely to be found in female travelers (86.7%) than in their male counterparts (13.3%). Conversely, males had a significantly higher percentage of vector-borne infections than females (89.6% vs. 10.4%). Juveniles (<20 years) often suffered from respiratory infections other than vector-borne infections. Vector-borne infections were more likely to occur in returning middle-aged Chinese travelers (97.9%) and less likely to be found in older travelers (2.1%). Moreover, vector-borne infections were more likely to be identified through a follow-up. Gastrointestinal infections were more often reported by on-board staff. By contrast, fever screening was sensitive to identifying respiratory infections and vector-borne infections.

**Figure 1:**
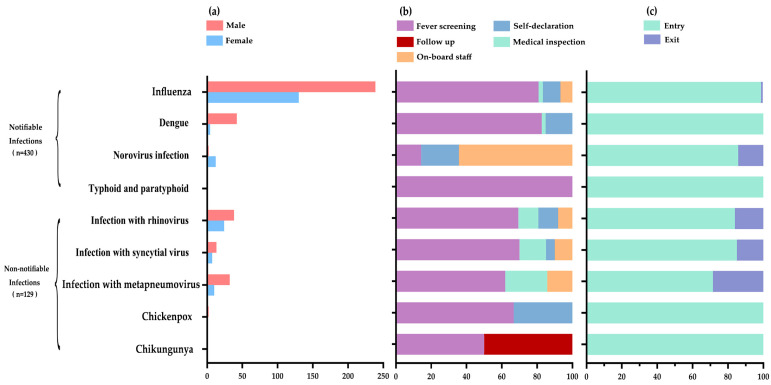
(**a**) The number of infection cases, (**b**) the proportion of case-screening approaches, (**c**) and the type of entry–exit port. Note: *y*-axis is the type of infectious disease, and *x*-axis is the number of cases.

Figure 1 shows the number of infections and the proportion of case-screening approaches. Nine types of pathogens were detected in the incoming passengers, among which four types were notifiable diseases (75.8%): influenza (369 cases), dengue (46 cases), norovirus infection (14 cases), and typhoid and paratyphoid (1 case). With the exception of norovirus infection, all other travel-related infectious diseases were more common in men than women. The most frequent notifiable infections were influenza and dengue. Moreover, fever screening detected the most notifiable infections. In contrast, norovirus infection was more often reported by on-board staff.

### 3.2. Temporal Patterns

**Figure 2:**
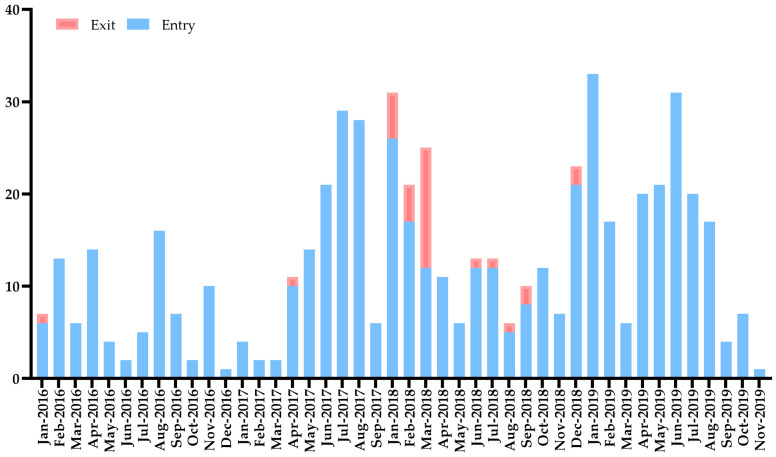
Temporal distribution of reported cases of infections at FOC Airport, China, 2016–2019. Note: *y*-axis is the number of cases, and *x*-axis refers to date.

Figure 2 shows the number of notifiable infections imported from overseas during the study period at FOC Airport. Travel-related infectious diseases were found throughout the year at the airport, and there were more entry-infectious cases in 2018–2019 (63.5%). Although the number of imported infections decreased slightly in 2018, the overall trend significantly increased during the 2016–2019 period (*p* < 0.001).

**Figure 3:**
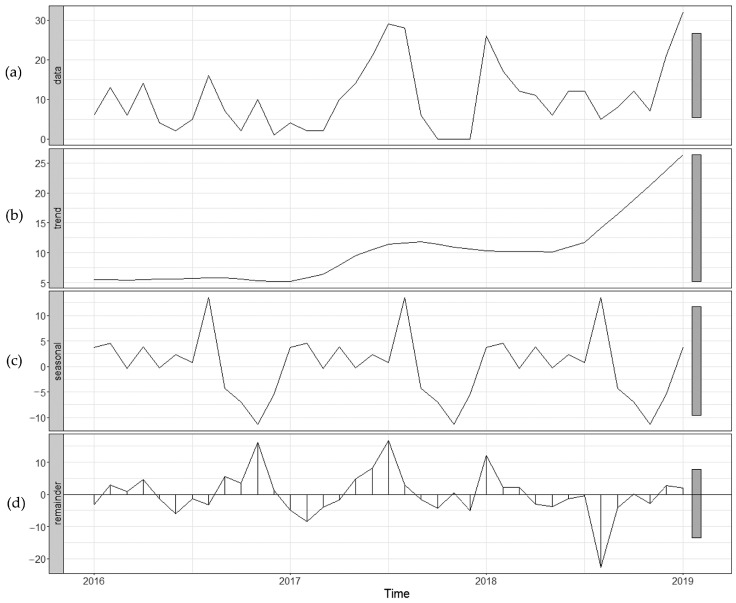
The seasonal decomposition based on STL of the imported infectious diseases at FOC Airport, China, 2016–2019. (**a**) Number of imported infectious diseases. (**b**) Secular trend component. (**c**) Seasonal component. (**d**) Remainder component.

**Figure 4:**
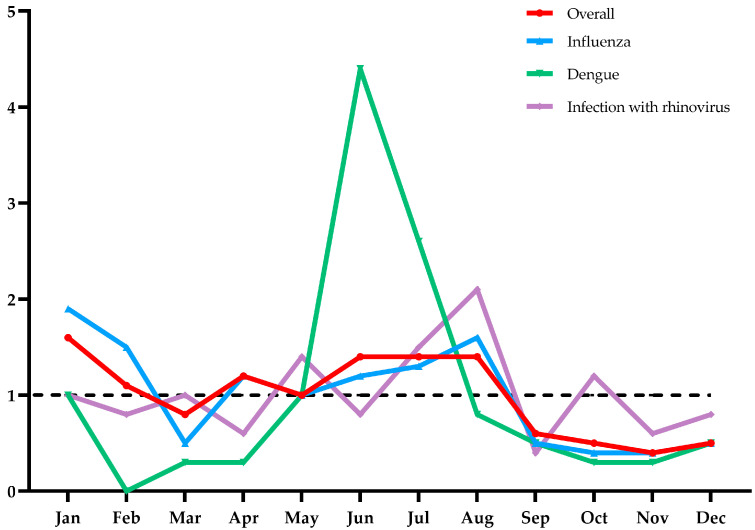
Seasonal index of the average monthly number of travel-related infections at FOC Airport, China, 2016–2019. Note: *y*-axis is the seasonal index, and *x*-axis refers to month.

Figure 3 and Figure 4 show the distribution of the seasonal characteristics of imported infectious diseases. On the whole, imported cases demonstrated a significant seasonal fluctuation trend, with peaks concentrated in January–February and May–August. And the seasonal fluctuation for dengue was more obvious than that for other infections.

### 3.3. Spatial Patterns

**Figure 5:**
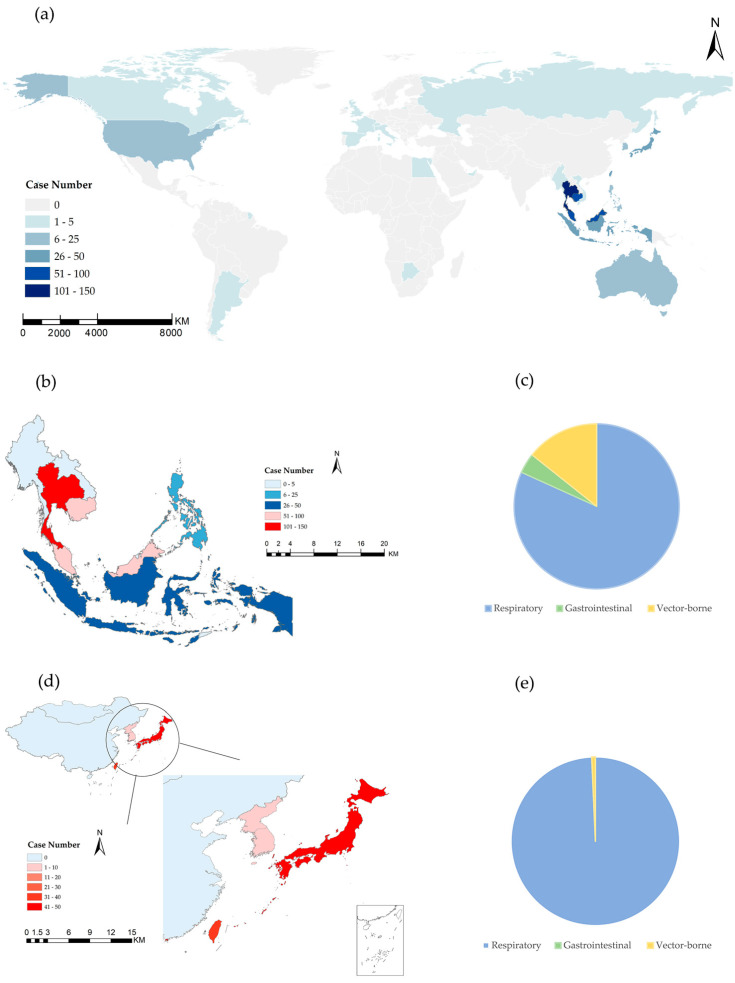
(**a**) Geographical distribution of imported infectious diseases. Distribution of imported diseases from (**b**) Southeast Asia and (**d**) East Asia. Distribution of types of imported diseases in (**c**) Southeast Asia and (**e**) East Asia.

Figure 5 shows the regional distribution of imported infections at FOC Airport during the 2016–2019 period. The major source areas of imported infections were identified as Southeast Asian countries (63.0%) and Eastern countries (27.0%) (a). Influenza cases accounted for more than half (60.3%) of the imported infections, with Thailand (23.8%) being the main country. Similarly, the majority of travelers with gastrointestinal infections were from Thailand (69.2%) (b and c). Among the imported infections in East Asian countries/regions, 99.3% were influenza infections, with Japan, Hong Kong, and Taiwan being the main regions from which the diseases were imported into mainland China.

All vector-borne cases were determined to be from Southeast Asian countries (97.8%), and only one dengue patient came from Hong Kong, China. Cases of influenza were predominantly diagnosed among the exit-travelers from China. There were no cases of vector-borne infectious cases among exit travelers.

### 3.4. Symptoms

As shown in Table 2, during the 2016–2019 period, of all the self-reported symptoms of travelers, the most frequently reported symptoms were fever (1913), cough (612), and runny nose (424). However, of the 1913 participants who reported having a fever, only 930 had a fever measured to be ≥37.8 °C. And fever was the common clinical symptom among patients with respiratory infections and vector-borne infections, while diarrhea was the main clinical symptom among patients with gastrointestinal infections, such as norovirus infection. Table 3 shows that fever was the common clinical symptom among patients with influenza and dengue fever, while diarrhea was the main clinical symptom among patients with norovirus infection.

## 4. Discussion

With the acceleration of globalization and increased population mobility, the spread of infectious diseases worldwide has intensified [15]. Frequent population movement significantly increases the risk of domestic notifiable infections, posing a serious challenge for infectious disease prevention and risk control. In this study, we analyzed the epidemiological characteristics of travel-related infections from abroad at FOC Airport from 2016 to 2019 and devised a regional infection spectrum of imported infections related to travel. The results of this study are helpful for better understanding the epidemic characteristics of imported infections and offer insights for the early detection and accurate prevention of travel-related infections.

In this study, we found that the number of imported symptomatic infection cases at FOC Airport increased during the study period, with most of them occurring in men aged 20–49 from Southeast Asian countries. This phenomenon reflects the fact that with frequent international exchanges, more and more Chinese individuals are living, working, and traveling abroad. Similar research results have also been reported in the existing literature [6,16]. Furthermore, imported infectious diseases exhibited distinct seasonality, with peaks observed in January–February and May–August. This seasonal pattern may be attributed to heightened international travel during holidays [17]. Traditionally, January and February coincide with the Chinese Spring Festival, while the months from May to August correspond to the summer vacation period for Chinese students. These periods are subject to a surge in overseas tourists returning to their hometowns, consequently leading to a transient increase in relevant infectious diseases.

Our findings underscore the importance of the continuous implementation of health-screening measures for international travelers. During the study period, 23.7% of travelers were identified as having travel-associated symptomatic infections, with 18.8% of the inbound travelers determined to have notifiable infections. While these numbers may potentially underestimate the efficacy of border-screening measures in identifying notifiable diseases, our results are supported by previous studies [7,18,19,20,21,22]. The detection of suspected infections was subject to the severity of a disease, the presence of clinical symptoms, and whether travelers had taken medication to escape health screening. Although customs quarantining may not be effective for all diseases, we assert that these measures are effective to a certain extent. At a minimum, they provide opportunities for raising awareness and educating the travelling public. In deciding whether to implement border-screening measures, authorities should consider the disease and outbreak characteristics, the country’s situation, the available resources, and the cost-effectiveness compared with other alterative measures. Studies have indicated that travelers are the most effective vessels for local transmission [23]. The border screening of travelers returning from endemic areas can provide complete epidemiological information on travel-associated infections. The implementation of border-screening measures and the timely implementation of relevant medical isolation measures for infected patients, especially those with clinical symptoms, can delay the entry time of cases and minimize the outbreak of local infectious diseases [24,25]. These measures have a positive effect on the prevention and control of imported symptomatic infections from abroad. Moreover, border health-screening measures may have a psychological impact that deters some travelers with infectious diseases.

In this study, we found that more than four out of five patients with influenza and dengue were detected 88.7% via fever screening. This suggests that fever screening is critical for the identification of suspected febrile travelers at ports of entry. In contrast, flight crew declarations were more sensitive for patients with gastrointestinal infections, as these infections are usually accompanied by obvious clinical manifestations such as diarrhea and vomiting. Interestingly, diarrhea affected 14 of 15 patients with enterovirus infection, attributed solely to norovirus infection. Norovirus infection is a common cause of diarrhea, especially among travelers. A German study found that norovirus infection was detected in 15.7% (*n* = 107) of travelers with diarrhea [26]. Another Southeast Asian study [27] found that 11.5% of 479 South Korean tourists with diarrhea were infected with norovirus. These findings are in line with our findings. However, diarrhea is different from other obvious clinical symptoms, and it is usually not easy for a crew to spot. Therefore, inbound tourists should be encouraged to strengthen their awareness of self-declaration and report their illnesses truthfully. In the meantime, crew members need to meticulously scrutinize the records of passengers’ self-screening questionnaires, pay attention to the physical condition of passengers, promptly report suspected cases, and instruct patients to seek medical treatment as soon as possible.

We found that influenza was the primary imported notifiable infection in this study. As a major hub for international travel and trade, China faces an elevated risk of influenza transmission from abroad. According to the China National Tourism Administration, the top three destinations (Thailand, Singapore, and Malaysia) for outbound tourism along China’s “Belt and Road” [28] zone align with the main source of influenza in this study. The increase in the number of tourists traveling to these countries may contribute to the introduction of imported cases. However, influenza outbreaks are not only caused by overseas-imported cases but also local cases. Imported influenza cases can be considered another important source of transmission for local outbreaks [2]. Therefore, in the prevention of local influenza outbreaks, concerted efforts should target imported cases, such as by promoting influenza vaccination among inbound travelers to minimize the risk of domestic transmission.

All the dengue infections identified in our study were imported, and they were predominantly among returning travelers from Cambodia. This may be attributed to the establishment of factories in Cambodia by Chinese businessmen in recent years, accompanied by the influx of migrant workers from China. Dengue fever is an imported infectious disease in mainland China, and it is a key factor triggering local outbreaks [29,30]. Sang et al. found that local dengue outbreaks in mainland China may have been caused by repeated imports from Southeast Asia [31]. Fujian Province, situated in the southeast coastal area of China, maintains close ties with Southeast Asian countries. It stands as one of the famous hometowns of overseas Chinese in China and one of the provinces most severely affected by dengue fever outbreaks. In recent years, the surge in global trade activities, escalating cross-border tourism, and the continuous export of this country’s domestic labor force has significantly contributed to the influx of imported dengue fever cases. Given the cost-effectiveness of interventions, more attention should be paid to the early identification of imported cases from other countries, especially Southeast Asian countries [30,32]. Specifically, the focus should be placed on the early identification and clinical management of imported cases of dengue fever in Southeast Asian countries, particularly with respect to improving the effectiveness of entry screening and strengthening the health monitoring and supervision of passengers traveling to and from dengue-fever-affected areas [29].

Our results show that imported dengue fever cases primarily occurred in the period from May to August. By contrast, the epidemic peak of dengue in Fujian was from August to October [33]. This temporal disparity suggests a time-lag relationship between imported and local dengue fever epidemics, and it can be attributed to the establishment of factories in Cambodia by Chinese businessmen in recent years, accompanied by the influx of migrant workers from China. For instance, Sang identified a 0–1 month lag effect between imported and local dengue fever cases in Guangzhou [34]. It should be noted that the data collected by Sang came from the local CDC, and there may be a time lag compared with the dengue cases screened at the port of entry. By contrast, Kuan analyzed the interaction between dengue cases screened at airport ports and local cases reported in Taiwan from 2007 to 2010. Their findings showed that there was a significant positive linear association between imported and local cases with a 1–3-month lag effect [35]. This is consistent with the results of our study. However, research exploring the potential interaction between imported cases detected at ports of entry and local cases remains scarce.

Moreover, multiple factors, such as temperature, rainfall, relative humidity, and the density of mosquitoes, have been implicated in dengue transmissions [36]. Mosquitoes serve as crucial vectors for dengue fever transmission [37,38]. The density of mosquitoes affects the occurrence and spread of dengue fever. Fujian has a subtropical monsoon climate characterized by high temperatures and a rainy summer, much like Southeast Asia, making it suitable for the survival and reproduction of Aedes albopictus [33,39]. The peak period of imported dengue fever in this study coincides with the active season of Aedes albopictus. Therefore, measures to prevent and control overseas-imported dengue fever should be intensified between May and August. Additionally, regular mosquito control efforts should be prioritized to curb the onset of dengue fever epidemics.

In this study, we examined the characteristics of imported infectious diseases. However, several limitations must be acknowledged. Firstly, we could not count asymptomatic patients who might not have been reported or suspected patients who might have taken medication to conceal their clinical symptoms. This may have resulted in low disease detection rates and a high number of missed diagnoses. Although the detection rates were a little bit low, border-screening measures could assist in the rapid triage of some symptomatic infections such as influenza and dengue cases that were in the viremic stage at the border and contribute to active sentinel surveillance. The blocking of the transmission of viruses from viremic travelers, including those with or without symptoms, to susceptible populations is critical to prevent outbreaks triggered by imported cases. Secondly, we only reported the proportion of infected cases by country, while the risk could not be calculated due to the lack of a denominator for the total number of inbound travelers. Additionally, our data only captured the trends of imported infectious diseases prior to the COVID-19 pandemic, overlooking the occurrence of entry infections occurring during or after the pandemic. Bai compared China’s statistics on notifiable infectious diseases reported during the COVID-19 outbreak in 2020 with those for the previous four years and found that a series of measures had been adopted by the Chinese government to prevent the spread of COVID-19. These measures, such as entry quarantine, wearing masks, and avoiding unnecessary gatherings, contributed to the containment of other notifiable infectious diseases to a certain extent [40]. DF is one of the diseases most significantly affected by the COVID-19 epidemic among all imported notifiable infectious diseases [41,42,43]. Nonetheless, the long-term impacts of these measures on dengue outbreaks remain largely unknown and warrant further investigation.

## 5. Conclusions

Controlling and preventing overseas-imported notifiable infectious diseases poses a growing challenge for public health authorities. Influenza and dengue fever are the most common infectious diseases imported into Fujian Province, with peak periods occurring in January–February and May–August. The majority of imported notifiable infectious diseases analyzed in this study originated from Southeast Asian countries. To effectively mitigate the risk of imported infectious diseases, stringent border controls and surveillance measures should be implemented to screen travelers for symptoms and provide necessary health declarations. Enhanced international cooperation and information sharing with other countries regarding outbreaks and trends may facilitate early detection and response measures. Multifaceted efforts including surveillance, vaccination, international collaboration, and public awareness are required to mitigate the importation and spread of infectious diseases from overseas sources.

## Figures and Tables

**Table 1:** Characteristics of imported infections identified at FOC Airport, China, 2016–2019.

	Overall * (*n* = 2362)	Not Detected * (*n* = 1803)	Respiratory ^#^ (*n* = 496)	Gastrointestinal ^#^ (*n* = 15)	Vector-Borne ^#^ (*n* = 48)
Gender					
Male	1508 (63.8)	1139 (63.2)	324 (65.3, −0.964)	2 (13.3, **−4.366**)	43 (89.6, **3.606**)
Female	851 (36.0)	661 (36.7)	172 (34.7, 0.964)	13 (86.7, **4.388**)	5 (10.4, **−3.606**)
Age (years)					
Median (IQR)	33 (0–96)	32 (0–96)	34 (0–34)	31 (2–68)	11 (23–51)
<20	853 (36.1)	700 (38.8)	151 (30.4, **4.573**)	2 (13.3, −1.236)	0 (0.0, **−4.448**)
20–49	1171 (49.6)	859 (47.6)	257 (51.8, **−5.343**)	8 (53.3, 0.196)	47 (97.9, **6.143**)
≥50	338 (14.3)	244 (13.5)	88 (17.7, 1.643)	5 (33.3, 1.734)	1 (2.1, **−2.854**)
Citizenship					
Mainland China	1896 (80.3)	1419 (78.7)	418 (84.3, **−1.98**)	12 (80.0, 0.592)	47 (97.9, **2.578**)
Hong Kong, Macau, or Taiwan regions	118 (5.0)	96 (5.3)	22 (4.4, 1.706)	0 (0.0, −0.795)	0 (0.0, −1.467)
Other countries	347 (14.7)	287 (15.9)	56 (11.3, 1.193)	1 (20.0, −1.175)	1 (2.1, **−2.025**)
Type of entry-exit port					
Entry	2249 (95.2)	1722 (95.5)	466 (94.0, 0.876)	13 (86.7, 1.333)	48 (100.0, 1.758)
Exit	111 (4.7)	80 (4.4)	29 (5.8, 0.876)	2 (13.3, 1.333)	0 (0.0, 1.758)
Case-finding approach					
Fever screening	1693 (717)	1268 (70.3)	383 (77.2, 1.848)	3 (20.0, **−5.153**)	39 (81.3, 0.886)
Medical inspection	124 (5.2)	94 (5.2)	29 (5.8, 1.413)	0 (0.0, −0.935)	1 (2.1, 1.056)
Self-declaration	261 (11.0)	205 (11.4)	46 (9.3, −1.643)	3 (20.0, 1.305)	7 (14.6, 1.102)
On-board staff	276 (11.7)	229 (12.7)	38 (7.7, −1.784)	9 (60.0, **7.299**)	0 (0.0, **−2.195**)
Follow up	2 (0.1)	1 (0.1)	0 (0.0, **−2.808**)	0 (0.0, −0.166)	1 (2.1, **3.266**)

Note: * refers to number and column percentages (in parentheses); ^#^ refers to number (column percentage, adjusted residuals). Cells with adjusted residuals greater than ±1.96 are highlighted in bold.

**Table 2:** Symptoms of imported cases of infections at FOC Airport, China, 2016–2019.

Symptoms	Overall (*n* = 2362)	No Virus Detected (*n* = 1803)	Respiratory (*n* = 496)	Gastrointestinal (*n* = 15)	Vector-Borne (*n* = 48)
Temp ≥ 37.8 °C	930	699 (75.2)	201 (21.6)	3 (0.3)	27 (2.9)
Cough	612	474 (77.5)	122 (19.9)	1 (0.2)	15 (2.5)
Runny nose	424	335 (79.0)	84 (19.8)	0 (0.0)	5 (1.2)
Nasal obstruction	58	43 (74.1)	16 (27.6)	0 (0.0)	0 (0.0)
Sore throat	43	37 (86.0)	6 (14.0)	0 (0.0)	0 (0.0)
Muscle aches	46	35 (76.1)	8 (17.4)	0 (0.0)	3 (6.5)
Sneezing	5	5 (100.0)	0 (0.0)	0 (0.0)	0 (0.0)
Diarrhea	94	65 (69.1)	15 (16.0)	12 (12.8)	2 (2.1)
Vomiting	132	102 (77.3)	26 (19.7)	1 (0.8)	3 (2.3)
Fever (self-reported)	1913	1479 (77.3)	393 (20.5)	3 (0.2)	38 (2.0)

**Table 3:** Symptoms of imported cases of notifiable infections at FOC Airport, China, 2016–2019.

Symptoms	Notifiable Infections (*n* = 423)	Influenza (*n* = 466)	Dengue (*n* = 46)	Norovirus Infection (*n* = 12)	Typhoid and Paratyphoid (*n* = 1)
Temp ≥ 37.8 °C	212	181 (85.4)	28 (13.2)	3 (1.4)	0 (0.0)
Cough	157	150 (95.5)	6 (3.8)	1 (0.6)	0 (0.0)
Runny nose	104	102 (98.1)	2 (1.9)	0 (0.0)	0 (0.0)
Nasal obstruction	13	13 (100.0)	0 (0.0)	0 (0.0)	0 (0.0)
Sore throat	3	3 (100.0)	0 (0.0)	0 (0.0)	0 (0.0)
Muscle aches	10	7 (70.0)	3 (30.0)	0 (0.0)	0 (0.0)
Sneezing	1	1 (100.0)	0 (0.0)	0 (0.0)	0 (0.0)
Diarrhea	15	2 (13.3)	1 (6.7)	11 (73.3)	1 (6.7)
Vomiting	8	4 (50.0)	1 (12.5)	3 (37.5)	0 (0.0)
Fever (self-reported)	382	335 (87.7)	44 (11.5)	3 (0.8)	0 (0.0)

## Data Availability

Data are contained within the article.
